# Global perspectives on the contribution of B cells to multiple sclerosis: an in-depth examination and evaluation

**DOI:** 10.3389/fimmu.2024.1442694

**Published:** 2024-11-14

**Authors:** Xinzhan Jiang, Hongyu Zhang, Yongtao Liu, Bo Sun, Guannan Mu

**Affiliations:** ^1^ Department of Neurobiology, Harbin Medical University, Harbin, China; ^2^ Department of Neurosurgery, Harbin Medical University, Harbin, China; ^3^ Biotherapy Center, Harbin Medical University Cancer Hospital, Harbin, China

**Keywords:** multiple sclerosis, B cells, neurodegeneration, CiteSpace, VOSviewer

## Abstract

**Background:**

Multiple sclerosis (MS) is a chronic, progressive autoimmune disease, with increasing attention on the role of B cells in its pathogenesis. Despite this growing interest, a comprehensive analysis of research trends and emerging foci on B cells in MS is currently lacking. In this research, we utilize a bibliometric approach to visualize and analyze research trends and focal points in this field, offering a valuable reference for future mechanistic studies in MS.

**Methods:**

We retrieved bibliometric data from the Web of Science Core Collection (WOSCC) for articles published between 2014 and 2023. VOSviewer 1.6.18 and CiteSpace 5.7R3 were used for co-authorship, co-occurrence, and citation analyses to identify key researchers, institutions, countries, and emerging themes in B cell research related to MS.

**Results:**

The analysis examined 5,578 articles published in 1,041 journals by 5,337 institutions globally. The United States leads in publication output, with Amit Bar-Or identified as the most influential author, and *Frontiers in Immunology* as the top journal in the field. Research has increasingly focused on the complex role of B cells in MS, particularly their involvement in the central nervous system (CNS) and mechanisms of anti-B cell therapy. Recent trends point to a growing focus on meningeal inflammation, kinase inhibitors, and Epstein-Barr virus, signaling a shift in research priorities.

**Conclusion:**

This bibliometric analysis highlights pivotal research trends, key contributors, and emerging areas of interest in B cell research in MS from 2013 to 2024. The findings underscore the growing recognition of the multifaceted role of B cells in MS pathogenesis, particularly their involvement in the CNS compartment and the potential of targeted therapies. The study identifies meningeal inflammation, Epstein-Barr virus infection, and kinase inhibitors as promising avenues for future research. The analyses driving the in-depth exploration of B cell mechanisms in MS and the development of novel diagnostic and therapeutic strategies provide researchers in the MS field with a comprehensive and objective perspective, serving as a valuable reference for accelerating the translation of basic research findings into clinical applications.

## 1 Introduction

Multiple sclerosis (MS) is a chronic, progressive autoimmune disease characterized by immune dysregulation, neuroinflammation, demyelination, and axonal degeneration (1–3). While the precise etiology of MS remains elusive, it is widely accepted that a complex interplay of genetic, environmental, and immunological factors contributes to its pathogenesis (4). Historically, MS was considered a T cell-mediated autoimmune disease, with CD4^+^ T helper (Th) 1 and Th17 cells being the primary effector cells responsible for the inflammatory process (5, 6). However, accumulating evidence suggests that B cells also play a crucial role in the initiation and progression of MS (7, 8).

B cells contribute to MS pathogenesis through various mechanisms, including antibody production, antigen presentation, and cytokine secretion (9). These cells can act as efficient antigen-presenting cells (APCs), presenting antigens to T cells and participating in the activation and maintenance of the immune response (10). Additionally, B cells secrete pro-inflammatory cytokines such as granulocyte-macrophage colony-stimulating factor (GM-CSF) and interleukin (IL)-6, as well as anti-inflammatory cytokines like IL-10, thereby modulating the pathological processes in MS (11).

The complex and multifaceted role of B cells in MS has been further highlighted by the emergence of new B cell subpopulations and the identification of novel mechanisms of action. The identification of GM-CSF-producing B cells has shed light on their contribution to the inflammatory milieu and their potential to promote T cell activation (12). The latest research found the imbalance of functional B cell subsets has been implicated in the pathogenesis of MS, with targeted inhibition of B-cell receptor (BCR) signaling pathways, mitochondrial respiration, or adenosine triphosphate (ATP) receptors showing potential therapeutic benefits (13).

Despite the growing recognition of the pivotal role of B cells in MS, a comprehensive analysis of the research landscape, trends, and emerging themes in this field is lacking. Bibliometric analysis is a powerful tool that integrates methodologies from information science, statistics, and network analysis to evaluate the impact, structure, and evolution of scientific research (14). By employing bibliometric techniques, researchers can identify key contributors, institutions, and research priorities, bibliometric methods have gained prominence for their ability to elucidate research trends and evaluate scholarly impact (15).

In this study, we conducted a bibliometric analysis of publications on B cell research in MS from 2014 to 2023, aiming to provide an objective and unbiased overview of the global research landscape. By employing visualization tools and methodologies, we performed co-authorship, co-occurrence, and citation analyses to identify influential researchers, institutions, countries, and research themes and provide researchers in the MS field with a comprehensive and objective perspective, serving as a valuable reference for accelerating the translation of basic research findings into clinical applications.

## 2 Materials and methods

### 2.1 Search strategies and dataset establishment

**Figure 1:**
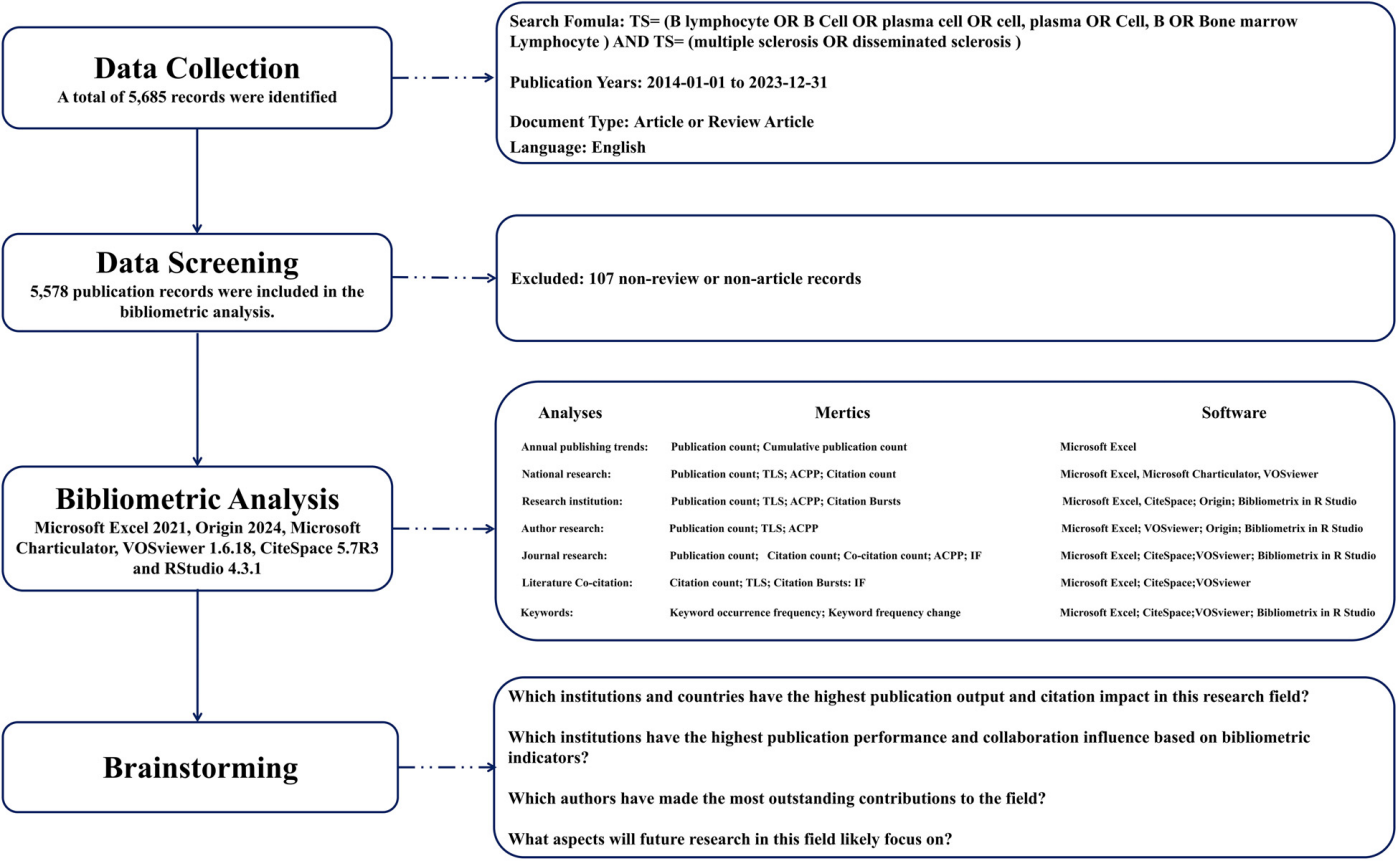
Methodological Framework for the Present Investigation. TLS, Total Link Strength; ACPP, Average Citation Per Publication; IF, Impact Factor.

The Web of Science (WOS), administered by Clarivate Analytics, is a prevalently utilized database for scientific literature (16, 17). It facilitates indexing and access to high-quality, peer-reviewed journal articles, conference proceedings, book chapters, and other scholarly works in the realm of scientific research (18). WOS encompasses a broad spectrum of disciplines including the natural sciences, social sciences, arts, and humanities, serving as a critical instrument for academic research, literature reviews, evaluations of scientific research, and scholarly tracking (19). In our investigation into the role of B cells in MS, we employed a comprehensive methodological approach. The search formula is: total search equation = (TS = (B lymphocyte OR B Cell OR plasma cell OR cell, plasma OR Cell, B OR Bone marrow Lymphocyte)) AND (TS = (Multiple Sclerosis OR disseminated sclerosis)). The inclusion criteria for source selection in this study are: 1) Manuscripts must clearly address the topic: the role of B cells in MS and be fully accessible, meaning that the full text of the article could be accessed either through the institutional subscriptions, open access, or other means that allowed full retrieval and analysis of the content; 2) Documents must be published between January 1, 2014 and 2023 December 31; 3) Only documents classified as “articles” or “reviews” were considered; 4) All publications must be in English. Exclusion criteria were: 1) Articles not directly related to the role of B cells in MS; 2) Other document types such as letters, reports, short communications, abstracts, etc.; 3) Repeated research. Two investigators independently assessed each publication for compliance with these criteria and consulted fully to ensure accuracy of data management. The methodical screening procedure is depicted in **Figure 1**. Through this systematic methodology, a total of 5,578 publications were retrieved from the WOS.

### 2.2 Bibliometric analysis and visualization

File information was downloaded from the Web of Science Core Collection (WOSCC) database, including full records and cited references in TXT format. A comprehensive bibliometric review and analysis were conducted on various facets of B cell involvement in MS, utilizing databases and analytical tools including Microsoft Excel 2021, Origin 2024, Microsoft Charticulator, VOSviewer 1.6.18, CiteSpace 5.7R3, and the Bibliometrix package within RStudio 4.3.1. VOSviewer was employed to identify the thematic clusters, using a unified approach to mapping and clustering bibliometric networks. The software generated cluster plots of highly co-cited references and keywords, presented through network, overlay, and density visualizations (20, 21). In this process, VOSviewer uses a technique known as visualization of similarities (VOS), which positions nodes (publications, authors, or keywords) based on their co-occurrence or co-citation relationships, creating a network map where related items are grouped into clusters (22). To identify these clusters, we first extracted keywords from the titles, abstracts, and keyword fields of the analyzed articles. These keywords were then used to construct a co-occurrence matrix, which records the frequency of two keywords appearing together in the same article. The more often two keywords co-occur, the stronger their relationship, which helps define the structure of the clusters. Once the matrix was built, VOSviewer applied its clustering algorithm to group related keywords into clusters. The cluster size was set with a minimum threshold of 105 keywords for each cluster in our analysis. This threshold was chosen to ensure that each identified cluster represented a significant thematic group within the literature. Lastly, network visualization was conducted, where colors indicate different clusters and node sizes correlate with keyword frequency. This procedure has been described in detail in previous publications (15).

Commonly utilized evaluation indicators in bibliometrics, such as publication count, citation counts, citation burst, etc., were selected to offer a comprehensive understanding of the research landscape, key contributors, and emerging trends within the field (23–25). Citation bursts, which represent sudden increases in the citation count of a particular publication or keyword over a specific period, are indicative of emerging research fronts or topics that have gained significant attention from the scientific community, with their burst strength determined using CiteSpace’s built-in Kleinberg algorithm (25, 26).

In our research, we utilized metrics for analyzing annual publishing trends include publication count, cumulative publication counts to evaluate overall research productivity and impact and can be processed by Microsoft Excel (23, 25). For national research analysis, metrics such as publication count, total link strength (TLS), average citations per publication (ACPP), and citation count are employed, utilizing tools such as Microsoft Excel, Microsoft Charticulator, and VOSviewer, to provide insights into global research trends and identify influential countries (27). In the analysis of research institutions, commonly employed metrics include publication count, TLS, ACPP, and citation bursts, analyzed using Microsoft Excel, CiteSpace, Origin, and Bibliometrix, which help to highlight key centers of expertise and the flow of knowledge (22). For author research, metrics such as publication count, TLS, and ACPP are analyzed using Microsoft Excel, VOSviewer, Origin, and Bibliometrix, enabling the identification of influential researchers and research groups (28). For journal research, metrics such as publication count, citation count, co-citation count, ACPP, and impact factor (IF) are typically analyzed using Microsoft Excel, CiteSpace, VOSviewer, and Bibliometrix, offering a comprehensive overview of the publication landscape and key dissemination channels (28). In literature co-citation analysis, metrics such as citation count, TLS, citation bursts, and impact factor are analyzed using tools such as Microsoft Excel, CiteSpace, and VOSviewer (29). Finally, in keyword analysis, metrics such as keyword occurrence frequency and keyword frequency change are examined using Microsoft Excel, CiteSpace, VOSviewer, and Bibliometrix, revealing evolving research themes and emerging topics within the field (30).

By combining these complementary metrics and analytical tools, we aimed to identify key trends, influential actors, and promising research directions, and generate a comprehensive and nuanced understanding of the research landscape, spanning bibliometric dimensions such as productivity, impact, collaboration, and thematic focus. Ultimately, this provides a valuable reference for the field of B cell research in MS.

In the visual network diagram, nodes symbolize various parameters such as country, institution, and keywords, with the node size indicating its relative significance. Diverse hues of lines connecting each node delineate the interactions among them, where line thickness mirrors the strength of the correlation between nodes; a thicker line denotes a more substantial connection.

## 3 Results

### 3.1 Analysis of annual publishing trends

**Figure 2:**
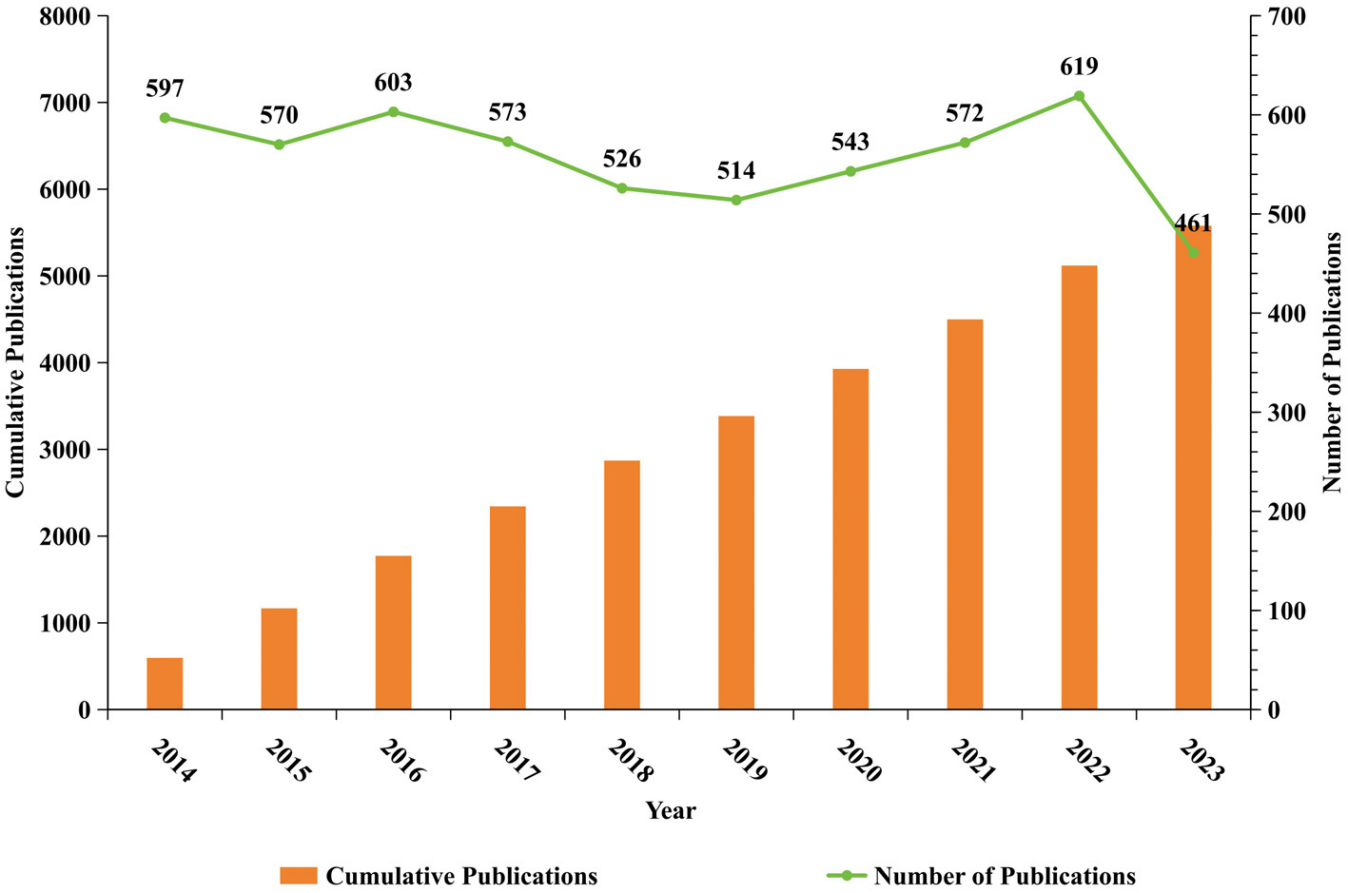
Annual publishing trends of B-cell-related scholarly publications in MS from January 1, 2014, to December 31, 2023. The orange bars in the figure represent the cumulative publications, while the green line represents number of publications. The horizontal axis represents the year, the left vertical axis represents the cumulative publications, and the right vertical axis represents the number of publications.

This bibliometric analysis evaluated 5,578 manuscripts on B cell research in the context of MS, spanning from 2014 to 2023, disseminated across 1,041 journals and originated from 5,337 institutions located in 102 countries, providing insights into publication trends and research impact over the decade. Analysis of Annual Publications from 2014 to 2023 (**Figure 2**). The data revealing fluctuations in research activity, after a peak of 603 publications in 2016, a temporary decline followed. However, publication volume rebounded steadily, reaching 619 articles in 2022, the highest in nearly a decade. In 2023, output sharply decreased to 461 articles, the lowest in the ten-year period from 2014 to 2023, the cumulative volume of publications steadily increased. By 2023, the total reached 5,578 articles. This trend suggests a steady upward trajectory in publication output, despite recent fluctuations, indicating a sustained interest in B cell research related to MS.

### 3.2 Analysis of national research

**Figure 3:**
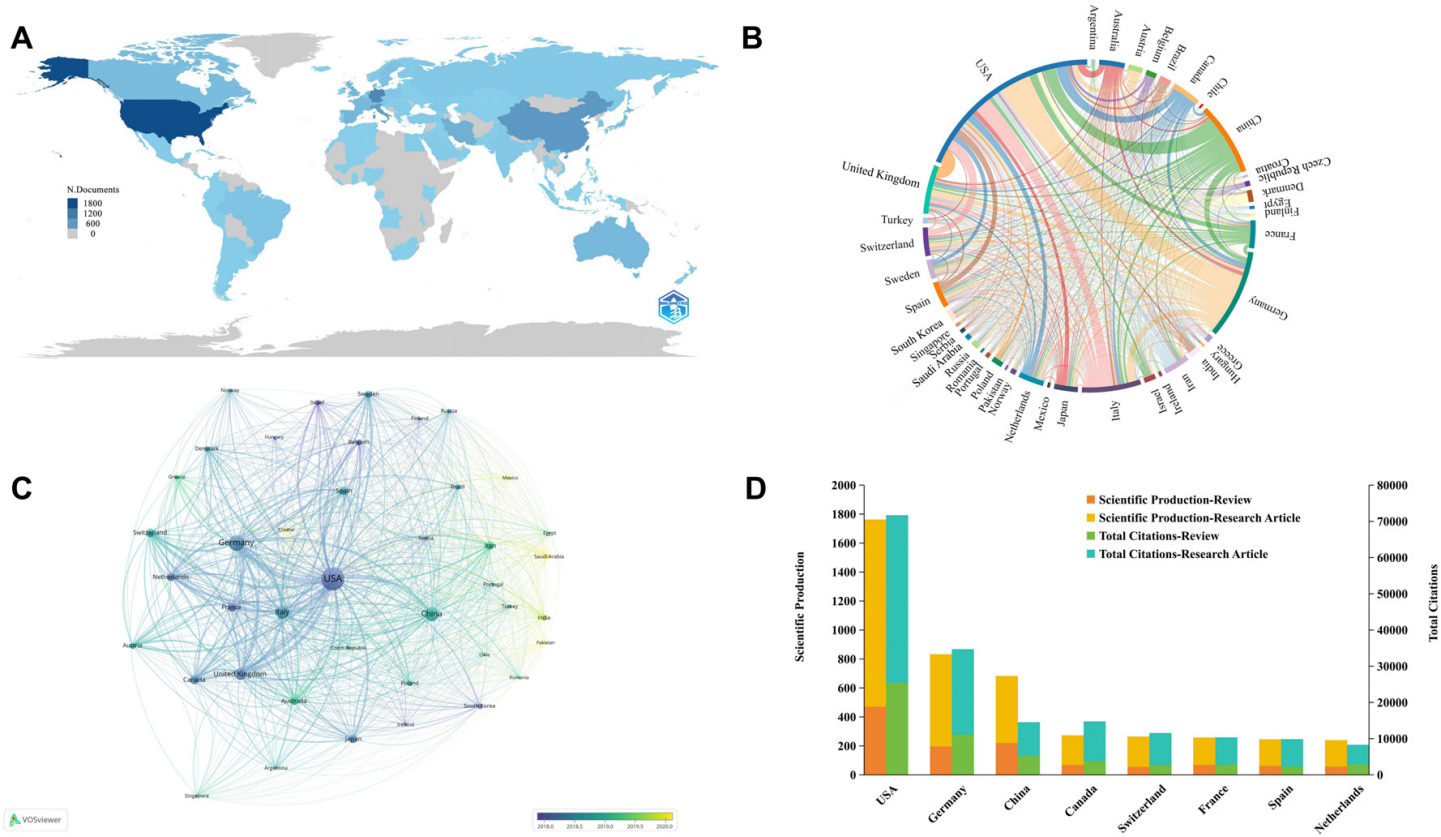
Global distribution and collaboration network of B cell research in MS from 2014 to 2023. **(A)** Global distribution of publications. **(B)** Chord diagram of cooperation relationships between countries. Line links indicate the existence of cooperation between countries. The thicker the lines, the closer the cooperation. **(C)** Network visualization of country collaborations. It shows countries with at least 20 published articles and the circle size indicates publication volume, while line thickness represents the strength of collaboration. **(D)** Top 10 countries by publication volume and citation categories.

This statistical analysis depicts the distribution and comparative assessment of B cells in MS research across countries over the past 10 years since 2014. During this period, research in this area involved 102 countries. Our results consistently show that the United States and Germany are the top contributing countries in terms of publication volume, citations, and international collaboration. As presented in **Table 1**, the United States leads with 31.59% of total publications, followed by Germany at 14.92%, while China ranks third at 12.23%. China ranks third globally in publication volume but has the lowest average citations among the top 10 countries, at 21.33%, compared to higher average citations for countries such as the United States (40.68%) and Germany (41.68%). The broad geographic distribution of publications across Europe, America, Asia, and Oceania (**Figure 3A**).

**Table 1:** Top 10 countries with the largest number of publications.

Rank	Regions	Counts	Citations	ACPP	TLS
1	United States	1,762 (31.59%)	71,679	40.68	2,086,202
2	Germany	832 (14.92%)	34,677	41.68	1,361,931
3	China	682 (12.23%)	14,547	21.33	535,527
4	Italy	551 (9.88%)	21,033	38.17	816,684
5	United Kingdom	455 (8.16%)	22,622	49.72	733,293
6	Canada	273 (4.89%)	14,742	54.00	513,398
7	Switzerland	264 (4.73%)	11,565	43.81	504,346
8	France	258 (4.63%)	10,384	40.25	389,255
9	Spain	245 (4.39%)	9,842	40.17	369,814
10	Netherlands	239 (4.28%)	8,287	34.67	399,539

ACPP, Average Citation Per Publication; TLS, Total Link Strength.

We conducted a visual analysis of international collaborations from 41 countries that each published more than 20 articles, to better understand the global research network in B cell research for MS (**Figure 3B**). The United States, Germany, and China emerged as major contributors to global research and key hubs for international scientific cooperation. These countries not only lead in publication volume but also in the establishment of significant research network. It’s noteworthy that while established research powerhouses continue to dominate, we observe emerging contributions from various regions. In Eastern Europe, countries such as Poland, Czech Republic have shown increased activity in this field. In the Middle East, Turkey, Iran, and Saudi Arabia have demonstrated growing research output. While these countries may not yet match the output of the leading nations, their growing participation suggests an expanding global interest in this research area (**Figure 3C**). Further research found that the United States leads in both research articles and review articles production, highlighting its comprehensive strength and quality in scientific research (**Figure 3D**).

### 3.3 Analysis of research institution

From 5,578 articles, we analyzed contributions from 5,537 institutions, identifying the top ten institutions by publication output over the past decade (**Table 2**). The University of California, San Francisco (United States), Karolinska Institute (Sweden), and Harvard Medical School (United States) were the leading contributors in B cell research related to MS, reflecting their pivotal role in advancing the field. The Medical University of Vienna has the highest ACPP, while the University of California, San Francisco ranks third but leads in total citations, followed by Harvard Medical School and the University of Pennsylvania, reflecting the significant contributions of the United States. Sun Yat-sen University is the only Chinese institution to appear in our ranking of the top 30 institutions in the world in terms of publication volume in related fields, placing 25th globally. This ranking underscore China’s role in the field and the potential for further growth in research contributions and collaborations. It also serves as an important bridge between China and other countries, fostering international collaborations in this field. (**Supplementary Table 1**).

**Table 2:** Top 10 institutions with the most publications.

Organization	Counts	Citations	ACPP	TLS	Location
University of California, San Francisco	101 (1.81%)	7, 437	73.63	277, 269	United States
Karolinska Institutet	100 (1.79%)	3, 960	39.60	200, 848	Sweden
Harvard Medical School	88 (1.58%)	6, 144	69.82	159, 771	United States
University of Sydney	84 (1.51%)	2, 208	26.29	164, 648	Australia
University of Tehran of Medical Sciences	80 (1.43%)	1, 441	18.01	104, 776	Iran
University of Pennsylvania	73 (1.31%)	5, 702	78.11	252, 629	United States
Medical University of Vienna	71 (1.27%)	5, 673	79.90	199, 786	Austria
Technical University of Munich	69 (1.24%)	4, 511	65.38	217, 474	Germany
Stanford University	66 (1.18%)	2, 345	35.53	117, 264	United States
Queen Mary University of London	64 (1.15%)	2, 940	45.94	149, 531	United Kindom

ACPP, Average Citation Per Publication; TLS, Total Link Strength.

**Figure 4:**
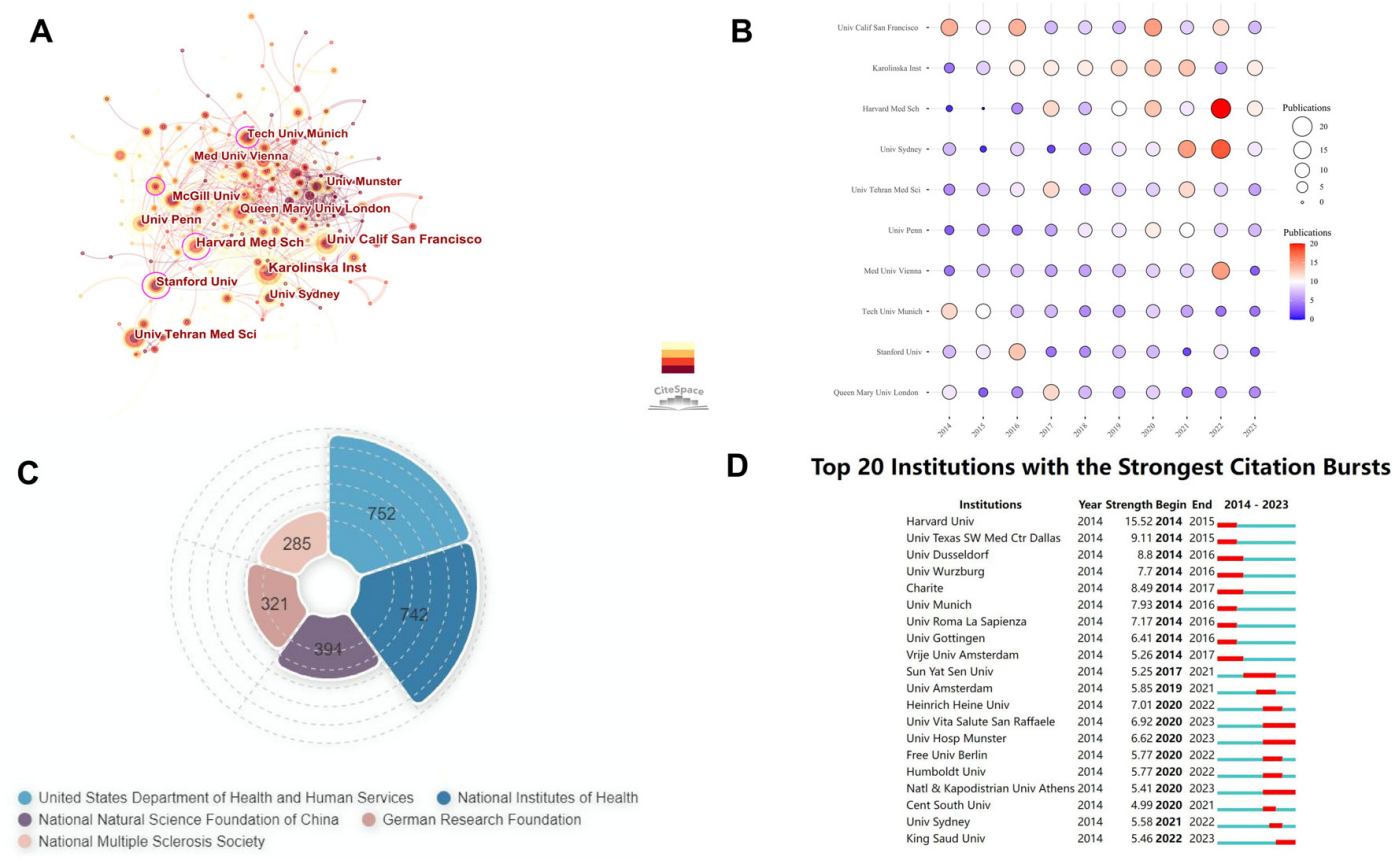
Institutional analysis of B cell research in MS from 2014 to 2023. **(A)** Institutional cooperation co-occurrence map. **(B)** Annual publication volume of the top 10 institutions. Larger circles and more intense colors indicate higher publication volumes. **(C)** Top 5 funding institutions by number of funded articles. The area size represents the number of funded articles. **(D)** Top 20 institutions with the strongest citation bursts.

Using CiteSpace, we analyzed institutional collaboration from 2014 to 2023 (**Figure 4A**). The dots represent research institutions, with lines indicating cooperative relationships. Dot size reflects the number of publications, and line thickness denotes the intensity of cooperation. Larger dots and thicker lines indicate higher publication volumes and stronger collaborations. Harvard Medical School is the most influential and collaborative institution. Analysis of the top 10 institutions’ annual publications shows the University of California, San Francisco led in 2014 with 14 articles, while Harvard Medical School had the least with 1 (**Figure 4B**). However, Harvard and the University of Sydney have shown growth, with Harvard publishing 20 articles in 2022. The observed increase in the number of publications supported by funding agencies may potentially account for this shift. (**Figure 4C**). The Technical University of Munich’s output declined, with only 4 articles in 2022 and 2023. We also examined institutions with significant citation increases from 2014 to 2023 (**Figure 4D**). Harvard University saw a substantial citation increase in 2014-2015, while Sun Yat-sen University gained attention from 2017 to 2021, with notable citation growth, reflecting its rising influence.

### 3.4 Analysis of author research

**Figure 5:**
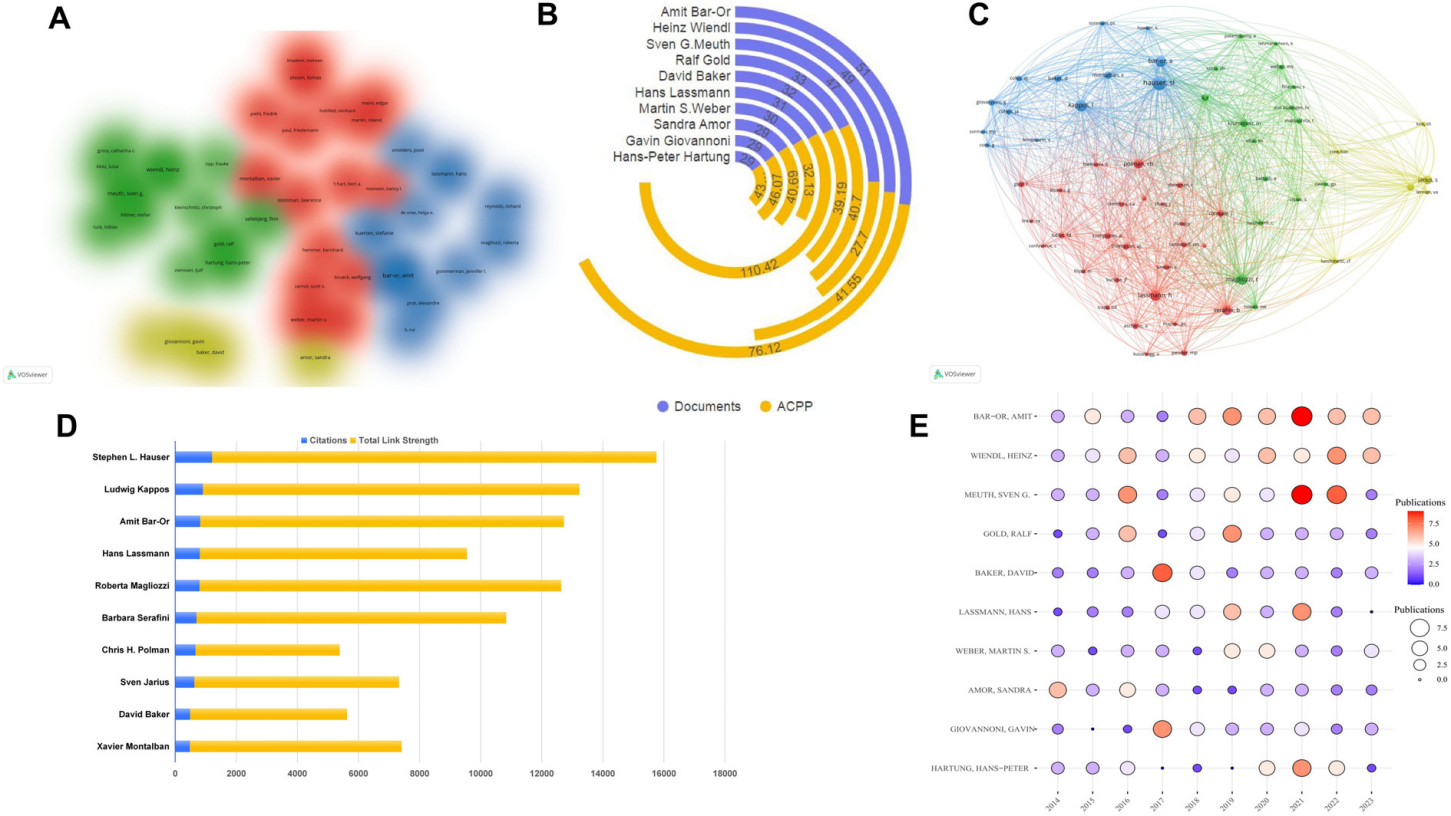
Author analysis of B cell research in MS from 2014 to 2023. **(A)** Cluster visualization of authors. The authors with at least 18 publications. Colors represent different research clusters or subfields within B cell research in MS, with each color denoting a group of authors who frequently collaborate or work on similar topics. **(B)** Number of publications of the top 10 authors. **(C)** Network visualization of most frequently cited authors. **(D)** The top 10 most cited authors. **(E)** Annual publication trends of the top 10 most published authors.

We analyzed 42 authors with at least 18 publications using VOSviewer software (**Figure 5A**). The data shows strong global cooperation, especially among authors from the United States and Germany, such as Amit Bar-Or, Heinz Wiendl, and Martin S. Weber. A focused analysis of the top 10 authors reveals concentration in the United States, Germany, and the United Kingdom (**Table 3**), underscoring the dominance of the United States in this field. Promoting international collaboration is crucial for the field’s sustainable development. Total citations measure a scholar’s impact, with Amit Bar-Or of the University of Pennsylvania being a notable example. Over the past decade, research on neuroimmune diseases from Amit Bar-Or, with a focus on MS, has yielded 51 publications and garnered 3,882 citations. The TLS, a metric quantifying impact within global knowledge networks (31), stands at 78,395, underscoring substantial contributions to the field. Bar-Or’s research team has made significant progress in B cell depletion therapy (BCDT). BCDT can effectively reduce the number of B cells and thereby reduce the inflammatory activity of MS by using anti-CD20 antibodies (such as rituximab, Ocrelizumab) and other methods (32, 33). In addition, the team is also committed to exploring various new B cell subpopulations and exploring specific B cell targets. They functionally redefined B cell subpopulations through cytokines and discovered MS pathogenic GM-CSF^+^ B cells, proposed for the first time that an imbalance of functional B cell subsets can lead to MS (12). The latest research shows that BCR-mediated mitochondrial respiration may be the root cause of pro-inflammatory B cell phenotype and functional abnormalities in MS patients (13). Although Amit Bar-Or has the highest total citations, the ACPP is lower than the ACPP of Hans Lassmann from Harvard Medical School, whose ACPP reaches 110.42 (**Figure 5B**). Lassmann’s research on brain lesions, blood-brain barrier damage, and follicular B cells in MS provides a foundation for further studies in the field.

**Table 3:** Top 10 authors with the most publications.

Rank	Author	Citations	TLS	Country	Institution	Research Focus
1	Amit Bar-Or	3, 882	78, 395	United States	University of Pennsylvania	Inadequate response; Rituximab
2	Heinz Wiendl	2, 036	35, 523	Germany	University of Munster	Therapeutic lymphocyte depletion
3	Sven G. Meuth	1, 302	24, 315	Germany	University of Wurzburg	B-cells; T-cells
4	Ralf Gold	1, 343	17, 268	Germany	Ruhr University Bochum	NF-κb; Oral BG-12
5	David Baker	1, 254	35, 314	United Kingdom	University of London	Blood-brain-barrier; T-cell responses
6	Hans Lassmann	3, 423	29, 598	United States	Harvard Medical School	B-cell follicles; Meningeal inflammation
7	Martin S. Weber	964	43, 505	Germany	University of Gottingen	Oligoclonal bands; Plasma-cells
8	Sandra Amor	1, 180	22, 922	United Kingdom	Queen Mary University of London	Epstein-barr-virus; Cerebrospinal-fluid
9	Gavin Giovannoni	1, 336	28, 216	United Kingdom	Queen Mary University of London	Anti-CD4 antibody; B-cells
10	Hans-Peter Hartung	1, 272	24, 416	Germany	Heinrich Heine University Dusseldorf	Lymphocyte depletion

TLS, Total Link Strength; NF-κB, Nuclear Factor kappa-light-chain-enhancer of activated B cells; BG-12, Dimethyl fumarate.

To understand knowledge structure and research trends, we conducted author co-citation analysis using VOSviewer and R studio. We analyzed 62 authors with over 200 citations each; the nodes represent authors, with node size corresponding to citation frequency and line thickness indicating the strength of co-citation links. Authors frequently co-cited are clustered together by color, reflecting intellectual collaborations and thematic research areas. This analysis uncovers tightly-knit author groups that drive key developments in MS research (**Figure 5C**). **Figure 5D** lists the top 10 most cited authors by co-citation analysis. Amit Bar-Or, while the top author, has 816 citations, less than Stephen L. Hauser, who has 1,208. Ludwig Kappos ranks second with 902 citations and a link strength of 12,328. The closely connected efforts are enhancing academic exchanges and knowledge dissemination. Analyzing the annual publication volume of the top 10 authors from 2014 to 2023 (**Figure 5E**; **Supplementary Table 2**). The publication trends of the authors demonstrate a notable rise in output, particularly between 2017 and 2020. This increase likely reflects the growing interest in B cell-targeted therapies and advancements in understanding B cell immunopathology. In 2021, Amit Bar-Or and Sven G. Meuth led the field with nine publications each, underscoring their active contributions to shaping current research directions.

### 3.5 Analysis of journal research

**Figure 6:**
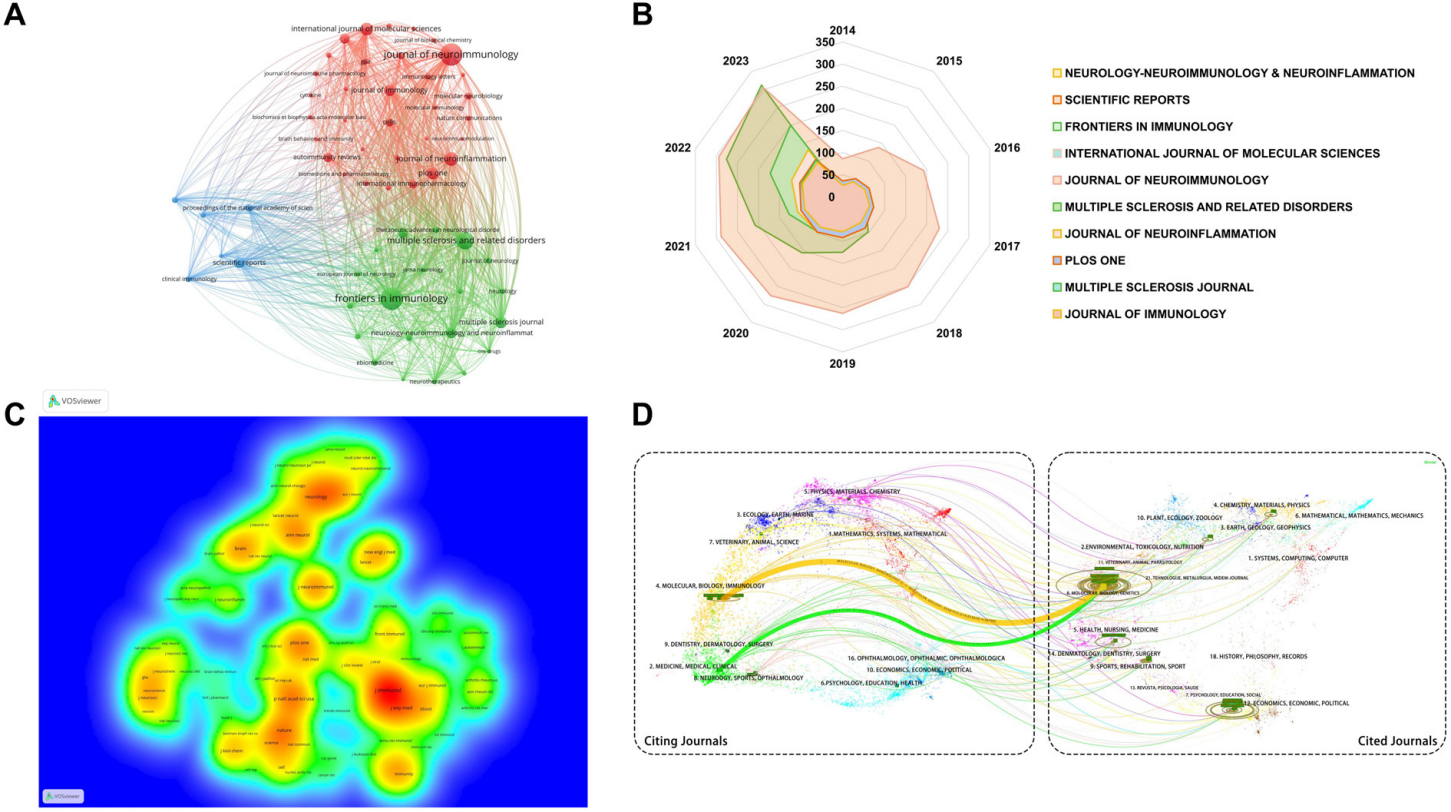
Journal research analysis of B cell research in MS from 2014 to 2023. **(A)** Cluster analysis of publications in journal. Colors signify different research themes within the field. Journals with the same color are more closely related in terms of their research focus or scope. **(B)** Annual cumulative publication volume of the top 10 journals. **(C)** Density map of co-cited journals. Denser areas indicate journals that are frequently cited together, forming research networks. Colors indicate the frequency of co-citation, warmer colors represent areas of high co-citation density, while cooler colors indicate lower co-citation frequency. **(D)** Dual-map overlay of citing and cited journal relationships. Dual-map overlay showing relationships between citing journals (left) and cited journals (right), with lines representing citation links.

Journal analysis plays a pivotal role in scientific research management, guiding academic publishing strategies and optimizing library resources. By evaluating the academic impact and quality of journals, it helps researchers and administrators identify key dissemination channels and assess research trends (34, 35). Over the past 10 years, 1,041 journals were analyzed. For a focused analysis, we selected 59 journals that published over 15 articles within the past decade, ensuring sufficient representation of impactful journals for visual cluster analysis (**Figure 6A**). This threshold allows for a comprehensive view of leading journals while filtering out those with limited publication output. Node size indicates publication volume, while lines show collaborative relationships among journals. Analysis the top 10 journals by publication volume (**Table 4**), the IF are widely recognized as a measure of journal influence, reflecting the average number of citations received by articles in a specific year (36). Quartile rankings provide an additional layer of evaluation, positioning journals within their field based on citation performance (37). Notably, while *Frontiers in Immunology* leads in publication volume and citations, the *Journal of Neuroinflammation*, despite publishing fewer papers, boasts the highest ACPP at 35.52, indicating that its publications attract significant scholarly attention and contribute high-quality research. These findings highlight substantial academic contributions and innovations within the analyzed journals.

**Table 4:** Top 10 Journals with the most publications.

Rank	Journal	Counts	Citations	ACPP	IF (2022)	Quartile in category
1	*Frontiers in Immunology*	312	8, 053	25.81	7.3	Q1
2	*Journal of Neuroimmunology*	306	6, 423	20.99	3.3	Q3
3	*Multiple Sclerosis and Related Disorders*	200	2, 889	14.45	4.0	Q2
4	*Journal of Neuroinflammation*	132	4, 689	35.52	9.3	Q1
5	*International Journal of Molecular Sciences*	117	2, 305	19.70	5.6	Q2
6	*Plos One*	104	2, 362	22.71	3.7	Q2
7	*Multiple Sclerosis Journal*	102	3, 054	29.94	5.8	Q1
8	*Journal of Immunology*	96	3, 360	35.00	4.4	Q2
9	*Neurology-Neuroimmunology and Neuroinflammation*	85	2, 006	23.60	8.8	Q1
10	*Scientific Reports*	84	1, 710	20.36	4.6	Q2

ACPP, Average Citation Per Publication; IF, Impact Factor.

Analysis of the annual publication output of the top 10 journals from 2014 to 2023 (**Figure 6B**) shows an overall upward trend. *Frontiers in Immunology* exhibited significant growth, while the *Journal of Neuroimmunology*’s expansion plateaued post-2018. Other journals saw steady increases in publication rates. **Table 5** highlights the top ten cited journals. The *Journal of Immunology* leads with 16,017 citations and an IF of 4.40 in 2022, primarily focusing on immunology. It is followed by *Neurology*, with 8,902 citations, and the *Proceedings of the National Academy of Sciences of the United States of America* (*PNAS*), with 8,883 citations. **Figure 6C** reflects these citation densities.

**Table 5:** Top 10 most co-cited institutions.

Rank	Co-Cited Journal	Citations	TLS	IF (2022)	Quartile in category
1	*Journal of Immunology*	16, 017	1, 053, 616	4.4	Q2
2	*Neurology*	8, 902	576, 505	9.9	Q1
3	*Proceedings of the National Academy of Sciences of the United States of America*	8, 883	621, 867	11.1	Q1
4	*Journal of Neuroimmunology*	8, 341	549, 598	3.3	Q3
5	*Annals of Neurology*	7, 988	536, 574	11.2	Q1
6	*Plos One*	7, 744	475, 080	3.7	Q2
7	*Brain*	7, 367	530, 350	14.5	Q1
8	*Nature*	7, 139	509, 776	64.8	Q1
9	*Journal of Experimental Medicine*	7, 086	516, 544	15.3	Q1
10	*Multiple Sclerosis Journal*	7, 037	458, 256	5.8	Q1

TLS, Total Link Strength; IF, Impact Factor.

**Figure 6D** presents a double graph overlay of journals citing and being cited in B cell MS research from 2014 to 2023. The left side shows citing journals, and the right side shows cited journals, with colored lines indicating citation paths. This visualization reveals interdisciplinary citation relationships, primarily in molecular biology, immunology, and clinical therapy. Most cited journals specialize in molecular biology and genetics, indicating a strong knowledge flow in these areas. The emergence of new journals and evolving subject trends have reshaped the academic landscape, exploring new research areas like sports science, materials science, chemistry, physics, and social sciences. The dual-image overlay analysis enhances understanding of the multidimensional relationships within these fields

### 3.6 Analysis of literature co-citation

**Figure 7:**
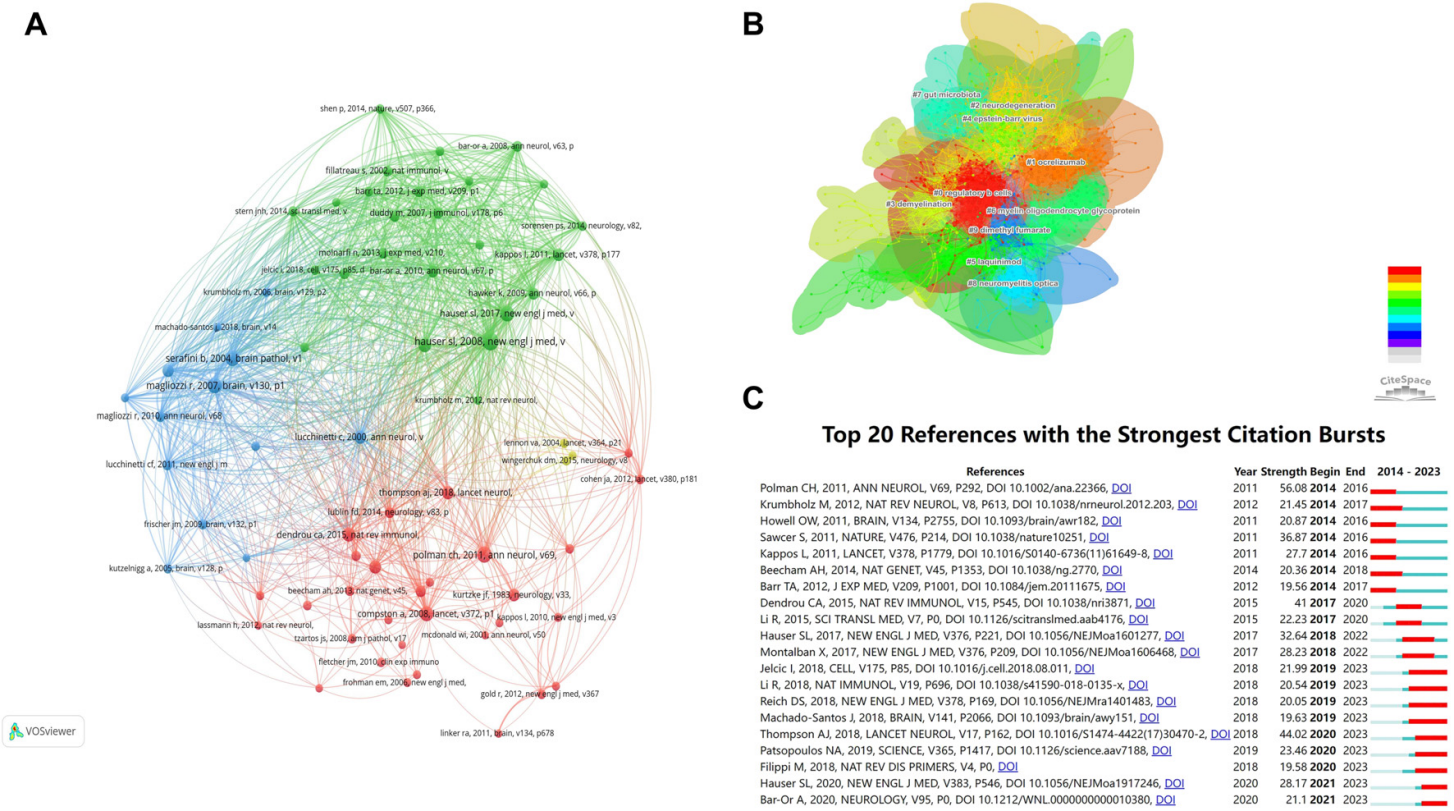
Co-citation analysis of B cell research in MS from 2014 to 2023. **(A)** Cluster analysis of cited documents. **(B)** Visualization and cluster analysis of co-cited documents. **(C)** Citation burst analysis of the top 20 most cited articles. The red line representing the duration of each article’s influence within the field.

Document co-citation analysis within the realm of bibliometrics represents a technique for investigating the interrelationships among documents (38). It facilitates the elucidation of the structural framework, evolutionary trends, and central themes within a research domain, offering scholars a macroscopic perspective on the knowledge architecture and developmental trajectories within the subject area (39) This approach aids in pinpointing research frontiers and prospective directions (39, 40). **Figure 7A** shows the overall structure of the co-citation network. The data shows that there are obvious clusters of cited documents among these co-cited documents.

Subsequent cluster analysis of co-cited documents identified distinct research orientations (**Figure 7B**). The main focus areas include regulatory B cells, ocrelizumab, neurodegeneration, demyelination, Epstein-Barr virus, laquinimod, myelin oligodendrocyte glycoprotein, gut microbiota, neuromyelitis optica, and dimethyl fumarate, highlighting the field’s knowledge structure and diversity. **Figure 7C** shows the twenty most frequently co-cited documents from 2014 to 2023, analyzing citation surges. Research focus shifts over time, influenced by emerging themes. Polman CH’s 2011 work in the *Annals of Neurology* experienced the highest citation surge from 2013 to 2016. Thompson AJ and colleagues’ 2018 update on MS diagnostic criteria in *Lancet Neurology* saw a notable citation increase from 2020 to 2023.

**Table 6** lists the top ten cited documents. Hauser S.L.’s “B-Cell Depletion with Rituximab in Relapsing–Remitting MS”, published in 2008 in the *New England Journal of Medicine*, ranks first with 469 citations and a TLS of 3,372. Magliozzi R’s 2007 paper on meningeal B-cell follicles in secondary progressive MS follows, indicating significant contributions to the field. The analysis shows that frequently co-cited authors are key contributors, aligning with authorship analysis findings.

**Table 6:** Top 10 most co-cited articles.

Co-cited reference	Publication year	Citations	TLS	IF (2022)	Quartile in category
B-Cell Depletion with Rituximab in Relapsing–Remitting Multiple Sclerosis	2008	469	3, 372	158.5	Q1
Diagnostic Criteria for Multiple Sclerosis: 2010 Revisions to the McDonald Criteria	2011	390	1, 211	11.2	Q1
Meningeal B-cell follicles in secondary progressive multiple sclerosis associate with early onset of disease and severe cortical pathology	2007	356	3, 002	14.5	Q1
Detection of Ectopic B-cell Follicles with Germinal Centers in the Meninges of Patients with Secondary Progressive Multiple Sclerosis	2004	348	2, 934	6.4	Q1
Ocrelizumab versus Interferon Beta-1a in Relapsing Multiple Sclerosis	2017	341	2, 489	158.5	Q1
Multiple sclerosis	2008	332	1, 196	168.9	Q1
Ocrelizumab versus Placebo in Primary Progressive Multiple Sclerosis	2017	307	2, 424	158.5	Q1
Immunopathology of multiple sclerosis	2015	275	1, 162	100.3	Q1
Diagnosis of multiple sclerosis: 2017 revisions of the McDonald criteria	2018	255	959	48.0	Q1
Heterogeneity of Multiple Sclerosis Lesions: Implications for the Pathogenesis of Demyelination	2000	253	1, 948	11.2	Q1

TLS, Total Link Strength; IF, Impact Factor.

### 3.7 Analysis of keywords

**Figure 8:**
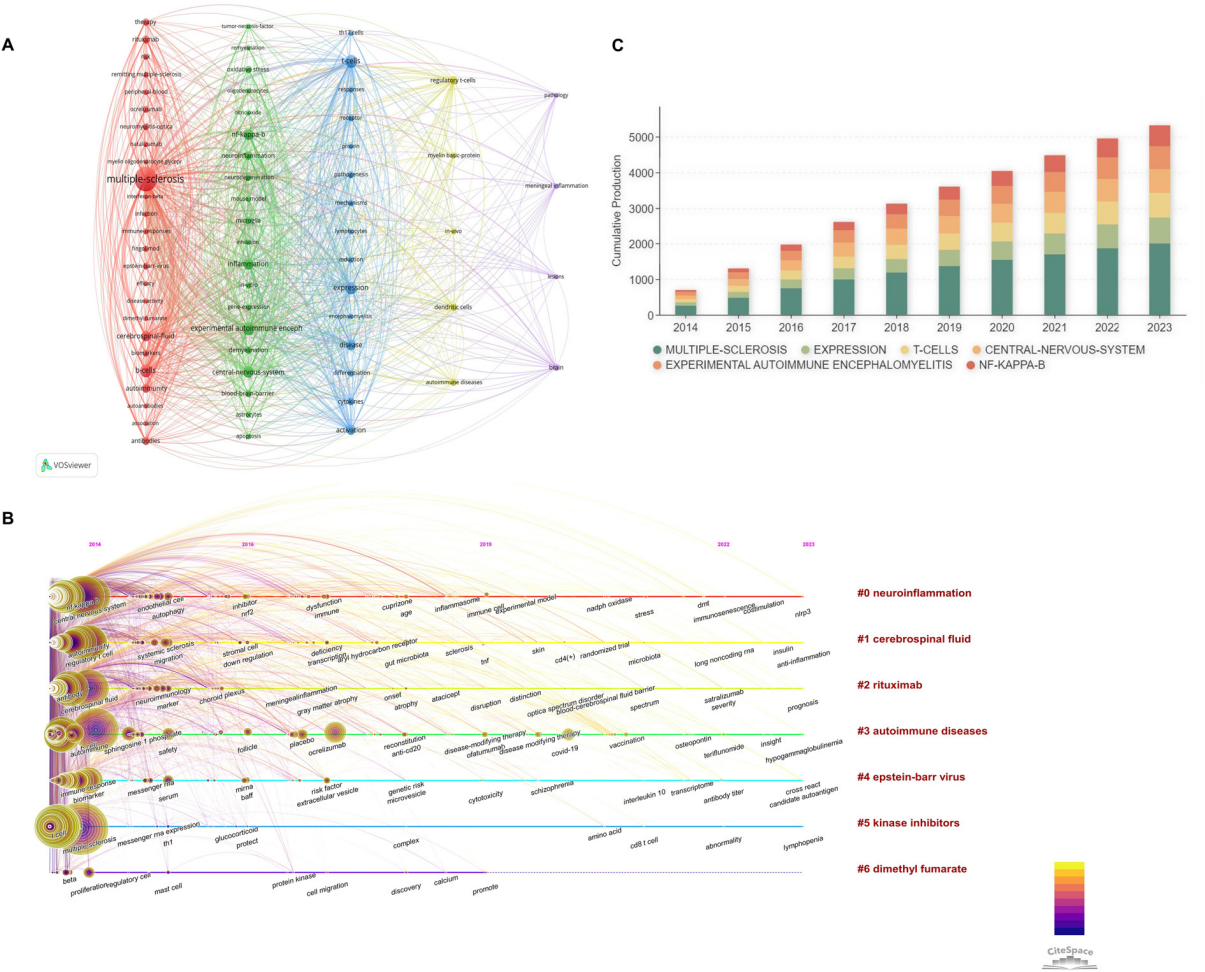
Keywords analysis of B cell research in MS from 2014 to 2023. **(A)** Cluster analysis of keyword. **(B)** Timeline visualization of keyword emergence and evolution. **(C)** Annual occurrence trends of the top 6 keywords.

Keyword analysis within bibliometric studies constitutes a pivotal methodology for delineating themes and discerning trends within scientific literature (41). Analyzing keywords from titles, abstracts, and keyword fields in articles from 2014 to 2023, we set a threshold of 105 occurrences, identifying 69 significant keywords. These were visualized through network overlays and density maps (**Figure 8A**), revealing five thematic clusters: The first cluster, delineated in red, encompasses terms such as “MS”, “therapy”, “rituximab”, “natalizumab”, among others, signifying a focus on clinical immunotherapy approaches. The second cluster, accentuated in green, centers on the study of animal models of disease, incorporating terms like “remyelination”, “experimental autoimmune encephalomyelitis”, “inflammation” and “central nervous system.” The third cluster, highlighted in blue, is dedicated to fundamental or mechanistic research pertinent to MS, featuring keywords such as “T cell”, “differentiation”, “responses”, “activation”, and “mechanisms”. The fourth cluster, the most pronounced in yellow, includes keywords “regulatory T-cells”, “myelin basic protein”, “dendritic cells”, and “autoimmune diseases”, indicating an exploration at the juncture of immunology and neuroscience, with a particular emphasis on autoimmune disease research. The fifth and final cluster, most prominent in purple, directs attention towards neuropathological studies, especially those concerning the brain, and includes “pathology”, “meningeal inflammation”, “lesions”, and “brain”. These clusters provide insights into the pathophysiology of neurological diseases, inform therapeutic strategies, and enhance diagnostic methodologies.

Temporal keyword analysis reveals that the earliest focus in this field was on T cells (**Figure 8B**). Over time, interest shifted towards B cells in MS, with anti-CD20 research becoming prominent around 2019. Rituximab and other anti-CD20 drugs emerged as key research targets. Targeting the B cell antigen CD20 has been shown to reduce the severity of the disease (42). However, since CD20 expression is limited to partially developed B cells, those in the early and terminal stages of maturation will persist following CD20 depletion (43). CD19-directed monoclonal antibodies emerged as a promising therapeutic approach, largely due to their extensive coverage of the B cell lineage (44). As a result, the field has increasingly focused on anti-CD19 therapy and the administration of anti-CD19 therapeutic agents, including Inebilizumab, can result in enhanced therapeutic outcomes (44). B cells can contribute to MS pathogenesis through their ability to present antigens to T cells, produce pro-inflammatory cytokines such as IL-6, and express unique markers such as CD20 (45). These findings underscore the importance of considering the diverse functions of B cells beyond antibody production when studying their role in MS. The global spread of COVID-19 in 2020 also influenced research directions, the impact of COVID-19 and associated vaccinations on MS has emerged as a prominent research focus. Recent research focuses include “neuroinflammation”, “cerebrospinal fluid”, “autoimmune diseases”, “Epstein-Barr virus”, “kinase inhibitors”, and “dimethyl fumarate”. Based on IF, citations, and keyword occurrences, six influential keywords were selected for annual analysis to explore research trends and shifts in academic interests (**Figure 8C**). Research in MS spans both clinical and fundamental studies, with significant attention to cytokine expression patterns. Increasing emphasis on “central nervous system” keywords highlights a shift from peripheral immunity to the central nervous system’s role in MS, reflecting its growing importance in academic research.

## 4 Discussion

MS is a chronic, progressive autoimmune disease that was initially regarded as a T-cell-mediated inflammatory response, with T cells playing a crucial role in the disease by producing inflammatory factors (46). The role of B cells in MS pathogenesis is complex and multifaceted, involving both peripheral and CNS-specific mechanisms (47). We conducted a comprehensive analysis of literature on B cell research in MS spanning from 2014 to 2023. Our analysis reveals that, despite a reduction in publication volume in 2019, there was a subsequent resurgence, reaching a peak in 2022. This trend demonstrates a sustained increase in research interest within this field.

The United States with the institutions in the United States notably the University of California, San Francisco, are at the forefront of publication output, underscoring its superior research resources. Researchers at the University of California, San Francisco have investigated the mechanisms by which B cells contribute to inflammation and demyelination, and explored the potential of B cell-targeted therapies, including rituximab and ocrelizumab, in modulating disease activity (32, 33). In addition to the pioneering work at the University of California, San Francisco, researchers at the Karolinska Institute in Sweden have made significant progress in elucidating the interaction between B cells and T cells in MS. They discovered that memory B cells can activate autoreactive, brain-homing CD4^+^ T cells, contributing to MS pathogenesis. This interaction presents a potential therapeutic target for disrupting the B cell-T cell axis in MS (48). At Harvard Medical School, researchers have linked Epstein-Barr virus to MS pathogenesis. Targeting memory B cells infected with the virus may offer a new therapeutic avenue, potentially leading to a cure for MS (49, 50).

Researchers from the United States, Germany, and the United Kingdom predominate in this field. Amit Bar-Or is a notable author with significant contributions to this field, and recent investigations concentrate on the involvement of B cell immune deficiency responses and the new B cell subpopulations in the pathology of MS (13). Bar-Or’s research shows imbalance of B cell subsets can lead to MS, targeting the B cell antigen CD20 to deplete B cells has been shown to reduce the relapse rate and slow the progression of disability in patients with MS (12, 51). Heinz Wiendl’s research has shown that Bruton’s tyrosine kinase (BTK) inhibitors, unlike anti-CD20 therapies, inhibit both B cell activation and myeloid cell function. This broader mechanism of action presents BTK inhibitors as a promising therapeutic strategy not only for MS but also for other autoimmune diseases and B cell malignancies (52). Hans Lassmann et al. found that BTK expression is associated with iron accumulation in myeloid cells in MS (53). Collectively, these pioneering efforts by researchers from institutions in the United States, Germany and other countries have significantly advanced our understanding of B cell involvement in MS pathogenesis, paving the way for novel therapeutic strategies aimed at modulating B cell activity and interactions within the CNS.

Assessing journals involves measuring both academic quality and influence. Key journals in this research domain include *Frontiers in Immunology*, *Journal of Neuroinflammation*, and *MS and Related Disorders*. *Frontiers in Immunology* stands as the preeminent journal in this field, boasting 312 publications and accruing 8,053 citations. The journal citation network highlights interdisciplinary connections and underscores the importance of cross-field collaboration and this analysis aids in the selection of publishing platforms and facilitates interdisciplinary research, thereby supporting effective research management.

Co-citation analysis delineates the knowledge framework and tracks the evolution of a given topic. Examining highly cited literature assists in comprehending the forefront of research. Hauser SL occupies a central position in the co-citation network, indicative of sustained influence. The article co-authored by Hauser SL and Amit Bar-Or, titled “B-Cell Depletion with Rituximab in Relapsing–Remitting MS”, remains the most co-cited publication over the past decade. The article initially posited that B cells could play a significant role in the pathogenesis of MS, it has been demonstrated that a single dose of Rituximab, which leads to B cell depletion, can reduce inflammatory lesions and clinical relapses within a 48-week timeframe (54).

Keyword analysis uncovers research areas including clinical immunotherapy and animal models and the convergence of immunology and neurology. The timeline analysis depicts the shift in research focus from T cells to B cells and highlights the changes in immunotherapy approaches.

Our bibliometric analysis has identified several emerging keywords that reflect important shifts in research priorities and areas of focus within the field. These include “meningeal inflammation”, “osteopontin”, and “Epstein-Barr virus”, among others. We believe that these trends have significant implications for advancing our understanding of the complex role of B cells in MS pathogenesis and for guiding future research efforts and therapeutic strategies. One of the most notable trends is the increasing focus on the role of B cells in the CNS compartment, as evidenced by the emergence of keywords such as “meningeal inflammation” and “cerebrospinal fluid”. This shift reflects a growing recognition of the importance of local B cell responses and their potential contribution to disease progression and neurodegeneration in MS. Recent studies have demonstrated the presence of ectopic B cell follicles in the meninges of patients with progressive MS, which are associated with increased cortical demyelination and neuronal loss (55, 56). These findings suggest that meningeal inflammation, driven in part by B cell infiltration and activation, may play a critical role in the pathogenesis of progressive MS (56, 57). As such, future research efforts should focus on elucidating the mechanisms by which B cells migrate to and populate the CNS compartment, and on developing targeted therapies that can modulate these processes. Another emerging trend is the increasing interest in the role of specific B cell-related molecules and pathways in MS, as highlighted by the keyword “osteopontin”. Osteopontin is a pleiotropic cytokine that has been implicated in various aspects of MS pathogenesis, including B cell activation and downregulate the co-stimulatory molecules CD80 and CD86 on B cell surfaces (58). Recent studies have shown that osteopontin levels are elevated in the serum and cerebrospinal fluid of MS patients, and that they correlate with disease activity and progression (59). These findings highlight the potential of osteopontin as a biomarker and therapeutic target in MS, and underscore the need for further research to elucidate its precise role in B cell-mediated pathology. Finally, the emergence of “Epstein-Barr virus” as a keyword reflects the growing recognition of the potential link between viral infection and MS pathogenesis, particularly in the context of B cell dysregulation (60). Epstein-Barr virus is a ubiquitous herpesvirus that infects and establishes latency in B cells, and has been associated with an increased risk of developing MS (51, 61). B cell disorders regulated by Epstein-Barr virus genes can result in the production of pro-inflammatory B cells (62). The recent research identified Epstein-Barr virus as a causative factor in MS, EBV-specific T cells are confirmed to play a key role in MS (50, 63). These findings highlight the need for further research to elucidate the mechanisms by which Epstein-Barr virus infection may promote B cell dysregulation in MS, and to develop targeted therapies that can modulate these processes.

The emerging keyword trends identified reflect important shifts in research priorities and areas of focus within the field of B cell research in MS. These trends highlight the increasing recognition of the complex and multifaceted role of B cells in MS pathogenesis, despite these advances, several knowledge gaps and challenges remain in the field of B cell research in MS. One major challenge is the lack of understanding of the precise mechanisms by which B cells contribute to the pathogenesis of the disease, particularly in the context of the complex interactions between B cells and other immune cell types, such as T cells and myeloid cells. Future studies should focus on elucidating these mechanisms using a combination of *in vitro*, *in vivo*, and *ex vivo* approaches, and on identifying key molecular pathways and targets for therapeutic intervention. Another challenge is the limited efficacy of current B cell-targeted therapies in the progressive forms of MS, particularly in the later stages of the disease. While anti-CD20 therapies have shown promise in reducing disease activity and slowing disability progression in some patients, there is a need for novel therapeutic strategies that can target the neurodegenerative processes and promote remyelination and repair. Future research should focus on identifying new B cell-related targets and developing combination therapies that can address both the inflammatory and neurodegenerative components of the disease.

The bibliometric analysis presented in this article comprehensively delineates the current trends and key areas of interest within the related research field, serving as a valuable reference for guiding future research endeavors. Nevertheless, there are certain limitations inherent in our research. Firstly, our investigation predominantly centers on the functions and roles of B cells in the context of MS. Our search strategy, while optimized to the best of our ability, may have overlooked some pertinent studies. The scope of our analysis was limited to select databases, focusing exclusively on English-language articles and reviews, potentially resulting in the exclusion of relevant data. Moreover, relying solely on publication and citation metrics can introduce biases that disadvantage certain groups, including women, researchers from low- and middle-income countries, and those working in less well-funded scientific disciplines (64, 65). The overrepresentation of male authors from high-income countries among the top-cited researchers in our analysis likely reflects systemic inequalities in opportunities and recognition rather than differences in scientific merit alone. Bibliometric indicators are imperfect proxies for research impact and quality, and they can perpetuate existing disparities. Future bibliometric studies in this field should strive to use a more diverse set of metrics, contextualize findings within broader structural factors, and provide a more holistic view of research impact. We encourage greater reflection within the MS research community on ways to create more equitable conditions for scientific work and recognition across genders, geographies, and disciplines.

Despite these limitations, our study provides valuable insights into the evolving landscape of B cell research in MS and highlights promising avenues for future investigation. By acknowledging the limitations and biases inherent in bibliometric analyses, we aim to present a balanced perspective and contribute to ongoing efforts to promote diversity, equity, and inclusion in scientific research.

## 5 Conclusion

This bibliometric analysis highlights pivotal research trends, key contributors, and emerging areas of interest in B cell research in MS from 2013 to 2024. The findings underscore the growing recognition of the multifaceted role of B cells in MS pathogenesis, particularly their involvement in the CNS compartment and the potential of targeted therapies. The study identifies meningeal inflammation, Epstein-Barr virus infection, and kinase inhibitors as promising avenues for future research. The analyses driving the in-depth exploration of B cell mechanisms in MS and the development of novel diagnostic and therapeutic strategies provide researchers in the MS field with a comprehensive and objective perspective, serving as a valuable reference for accelerating the translation of basic research findings into clinical applications.

## Data Availability

The original contributions presented in the study are included in the article/**Supplementary Material**. Further inquiries can be directed to the corresponding author.
